# Hereditary Hemorrhagic Telangiectasia with Unusual Associations

**DOI:** 10.7759/cureus.278

**Published:** 2015-06-16

**Authors:** Dheeraj Jain, Stalin Viswanathan, Chandramohan Ramasamy

**Affiliations:** 1 Department of General Medicine, Indira Gandhi Medical College & Research Institute; 2 Department of Cardiology, JIPMER

**Keywords:** hereditary hemorrhagic telangiectasia, pulmonary hypertension, calcinosis cutis, interstitial lung disease

## Abstract

We describe a report of an elderly lady who was hospitalized with progressive worsening of breathlessness and fatigue of one month's duration. Clinical evaluation of the patient revealed hereditary hemorrhagic telangiectasia, interstitial lung disease, pulmonary hypertension without left heart failure, and bilateral gluteal calcinosis cutis. Initially, CREST (calcinosis, Raynaud's phenomenon, esophageal dysmotility, sclerodactyly, and telangiectasia) syndrome was considered in view of the telangiectasia and calcinosis cutis, but a strong autosomal inheritance pattern and endoscopies (nasal and upper gastrointestinal) favored a diagnosis of hereditary hemorrhagic telangiectasia with rare associations.

## Introduction

Hereditary hemorrhagic telangiectasia (HHT, Osler-Weber-Rendu syndrome) is characterized by a classic triad of mucocutaneous telangiectasia, arteriovenous malformations (AVM), and autosomal dominant inheritance. Telangiectasias are nearly universal, but other lesions, such as AVMs, appear to be frequent only in certain forms of HHT. Spontaneous recurrent epistaxis due to nasal mucosal telangiectasia is the most common clinical manifestation of HHT [1].

Pulmonary hypertension develops in these patients generally secondary to high output failure that is related to liver vascular malformations [2]. Pulmonary AVMs are often multiple and bilateral, with a predilection for the lower lobes. Fifteen to 45 percent of patients with HHT have pulmonary AVMs, but the incidence of these lesions varies according to the specific gene that is present (in descending order of incidence: endoglin mutation [ENG-HHT1], activin receptor-like kinase mutation [ACVRL1-HHT2], and SMAD4 mutation) [2-3]. 

## Case presentation

Signed informed patient consent was obtained for this patient's treatment. No patient identifying data was included in this paper.

This 65-year-old lady presented with easy fatigability and breathlessness on exertion for the last 15 years that had worsened over the preceding month. She had a history of recurrent epistaxis since childhood and of melena intermittently. There were no other bleeding abnormalities. She had been hospitalized on multiple occasions and had received several blood transfusions for iron deficiency anemia. Eight years ago, abdominal ultrasonography and echocardiography had been normal. Erosive gastritis had been reported on esophagogastroscopy during that admission.

**Figure 1:**
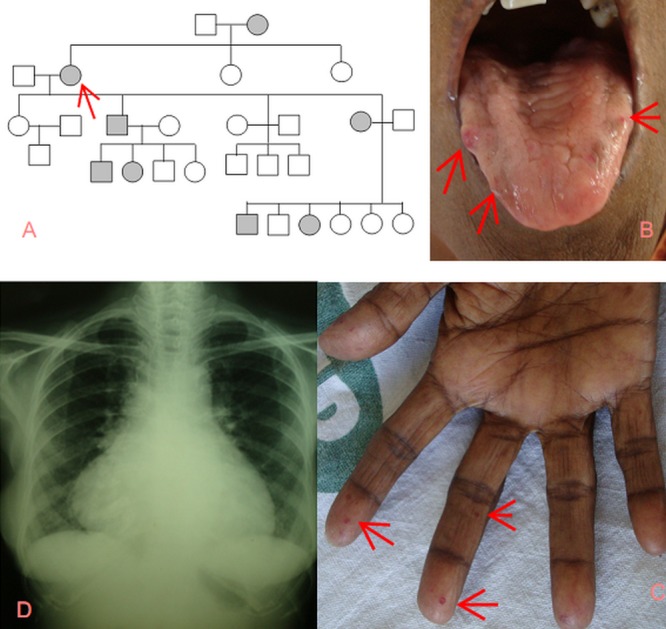
Family history and symptoms 1A: Family pedigree revealing autosomal dominant pattern; 1B: Telangiectasia on the tongue; 1C: Telangiectasia in the fingers; 1D: CXR –cardiomegaly and bilateral lower-zone haziness

Family history revealed epistaxis among first-degree relatives (Figure 1A). Her mother had died at 37 years following a bout of hematemesis. On examination, severe pallor, glossitis, and elevated jugular venous pressure were seen. Telangiectasias were observed on the fingers, tongue, and buccal mucosa (Figures 1B-1C). Auscultation revealed a loud P2, an ejection systolic murmur, and bilateral end-inspiratory Velcro crepitations. The rest of the systemic examination was non-contributory.

**Figure 2:**
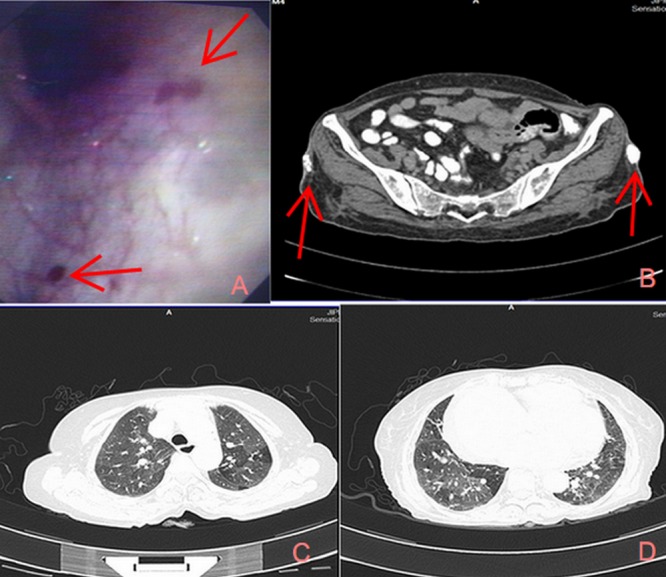
Investigations 2A: Telangiectasia in the gastric mucosa; 2B: CT abdomen revealed bilateral gluteal skin; 2C-2D: CT thorax - bilateral ground-glass appearance suggestive of interstitial lung disease

Investigations revealed a hemoglobin of 4.8 g/dl with a microcytic hypochromic blood picture, normal renal and liver function tests, negative stool for occult blood, cardiomegaly on chest radiography (Figure 1D), right atrial and ventricular enlargement on echocardiography with a RVSP of 55 mmHg, and esophageal and stomach telangiectasias on esophagogastroscopy (Figure 2A). Thoracic and abdominal computed tomography did not reveal any AV malformations; ground-glassing in both the lower lung lobes and bilateral gluteal skin calcifications (Figures 2B-2D) were found.   

She was not willing to undergo a gluteal skin biopsy. The patient also could not afford testing for anti-topoisomerase 1 antibody. A definite diagnosis of hereditary hemorrhagic telangiectasia was made with 3/4 of the Curacao criteria being satisfied [5]. The patient did not have an upper gastrointestinal bleed during her hospital stay, and her stool occult blood remained negative. She improved with RBC transfusion and was discharged from the hospital with advice to continue iron supplements, diuretics, and sildenafil. Fourteen months later, she was doing well on follow-up, with her haemoglobin having improved to 10.5 g/dL.  

## Discussion

Curaçao criteria, established by the HHT Foundation International, are useful for clinical diagnosis. Three or more among the four criteria suggests a definite clinical diagnosis of HHT; a probable diagnosis is considered in the presence of two criteria. The criteria include 1) spontaneous recurrent nose bleeds, 2) mucocutaneous telangiectasia, 3) visceral AVMs, and 4) an affected first-degree relative [4]. An unlikely diagnosis is made if ≤ 1 criterion is present. In patients with an ‘unlikely’ diagnosis, gene analysis may rarely show mutations (ENG, ACVRL1, or SMAD4), and hence, false negatives may be rarely observed with Curaçao criteria [5]. Pulmonary hypertension is rare in HHT. It is generally due to systemic arteriovenous shunting in the liver, which increases cardiac output, or it may be clinically and histologically indistinguishable from idiopathic pulmonary artery hypertension [2]. In our patient, pulmonary hypertension was probably contributed to by interstitial lung disease (clinical and radiological) since there was no evidence of AVMs on thoracic imaging.

There are no previously published reports of interstitial lung disease (ILD) in HHT. Rheumatoid arthritis with lung involvement has been described in association with HHT on one occasion in a 33-year-old man [6]. The lung manifestations in that patient had been controlled with steroids. It is also apparent that new vascular malformations continue to evolve, as evident in this case - a negative esophagogastroscopy eight years ago, now revealing typical telangiectasia (Figure 1C). In one-fifth of patients with HHT, gastrointestinal telangiectasia occurs in older individuals (after the third to fourth decade), compared to skin telangiectasia that develops by 30 years of age [3].

Apart from incidentally observed calcinosis cutis and telangiectasia in our patient, other features of the CREST syndrome or scleroderma were not seen. This association of HHT with calcinosis cutis has been reported only once earlier, in an elderly Japanese female who presented with a finger nodule prior to her diagnosis [7]. Association of genes encoding nitric oxide synthase and transforming growth factor (TGF) β have been shown in systemic sclerosis [8]. Altered signalling of TGF-β and activity of endothelial nitric oxide synthase have been uncovered in HHT [2].

## Conclusions

HHT is a rare disorder, and we describe one such patient who also had interstitial lung disease (hitherto unreported), pulmonary hypertension without left heart failure, and calcinosis cutis (second instance). Even though reasons for symptoms, such as dyspnea, are obvious, unusual causes like ILD, as in our case, may spring a surprise. Whether HHT is related to autoimmune diseases like rheumatoid arthritis and scleroderma is not known. Routine serological testing for autoimmune antibodies may probably be required in patients with HHT.
